# Role Mismatch in Medical Decision-Making Participation Is Associated with Anxiety and Depression in Family Members of Patients in the Intensive Care Unit

**DOI:** 10.1155/2022/8027422

**Published:** 2022-04-16

**Authors:** Tingting Fang, Pengfei Du, Yin Wang, Dandan Chen, Hailin Lu, Haoran Cheng, Wenqing Hu, Donghui Jiang

**Affiliations:** ^1^Department of Intensive Care, Affiliated Hospital of Jiangnan University, Wuxi 214122, China; ^2^Wuxi School of Medicine, Jiangnan University, Wuxi 214122, China

## Abstract

This study aimed to investigate the mismatch between the preferred and actual roles in the medical decision-making of intensive care unit (ICU) patients' family members and the relationship between the role mismatch of family members' decisions and anxiety and depression syndromes. A total of 223 family members of ICU patients in the Affiliated Hospital of Jiangnan University in China were enrolled. The simple Chinese version of the Control Preference Scale was used to complete the surveys to assess the preferred and actual roles, and anxiety and depression syndromes were measured using the Generalized Anxiety Disorder-7 scale and Patient Health Questionnaire-9, respectively. For the preferred and actual roles, the active role rates were 16.1% and 8.1%, the cooperative role rates were 49.3% and 31.4%, and the passive role rates were 34.5% and 60.5%, respectively. The incidence of mismatch was 43.0% between the preferred and actual roles, and the consistency between their preferred and actual decision-making roles was poor (kappa = 0.309, *P* < 0.001). Family members with mismatched decision-making roles had significantly higher incidence rates of anxiety (90.6% vs. 57.5%, *P* < 0.001) and depression (86.5% vs. 63.0%, *P* < 0.001). Logistic regression analysis revealed that mismatches in decision-making roles remained independently associated with these outcomes after adjustment for family members' sociodemographic features. The results of the present study demonstrate that the preferred role of ICU patients' family members is mainly cooperative, and the actual role is mainly passive. The mismatch between the preferred and actual roles is associated with anxiety and depression among the ICU patients' family members.

## 1. Introduction

Patients admitted to the intensive care unit (ICU) have various types of acute and critical illnesses and injuries, including multiple organ system dysfunction, and their conditions are peculiar, complex, and variable [1, 2]. The vast majority of ICU patients lose their ability to participate in medical decisions due to the severity of their condition, and patients who are incapacitated generally rely on surrogate decision-makers (usually family members) to make necessary medical decisions [3, 4].

With the development of society and the improvements of human rights awareness, the willingness of patients and their families to participate in medical decisions has been increasingly affected [5]. It has been reported that two-thirds of ICU patients' family members wish to participate in medical decision-making [6]. The active medical decision-making participation of family members strengthens doctor-patient communication, reduces family members' anxiety, depression, and other adverse psychological reactions; and shortens patients' duration of stay in ICU [7]. However, ICU patient families commonly take on a passive role in decision-making participation during the actual decision-making process in China [8], unlike the role of cooperative decision-making in Canada [9].

Psychological symptoms, such as anxiety and depression, are relatively common among family members of patients in the ICU [10]. It is a challenging process for ICU patients' family members to participate in medical treatments as alternative decision-makers, which is significantly related to the morbidity of psychological symptoms [11, 12]. If the preferred decision-making role of ICU patients' family members does not match the actual decision-making role, a significant increase in psychological symptoms of depression occurs [13]. Previous studies have shown that the mismatch between preferred and actual decision-making roles is associated with increased patient anxiety [14], and this condition may also be present in patients' family members [3]. In addition, the presence of psychological symptoms in ICU patients' family members was reported to have a negative impact on their daily living activities, their support and care of the patients [15], and their participation in medical decision-making [12].

Currently, there are few studies on the medical decision-making participation of ICU patient's family members in China. We conducted a survey to examine the preferred and actual role of ICU patients' family members in decision-making and to explore the relationship between decision-making role mismatch and the development of anxiety and depression in patients' family members.

## 2. Materials and Methods

### 2.1. Study Subjects

This study was conducted in the Affiliated Hospital of Jiangnan University using the convenient sampling method, and 223 family members of patients who were treated in the ICU from October 2020 to April 2021 were selected as the study subjects. To maximize recruitment, we defined the ICU patient as an individual who stayed in the unit for more than 24 hours and had no decision-making ability, and we included only one family member for each patient [8]. The family member who participated in the study was at least 18 years old and served as decision-makers during the patient's treatment [8]. The family member was required to have normal understanding and be able to communicate in Mandarin Chinese. Family members were excluded if they had experienced severe mental illness, psychological trauma, or hearing/language expression disorders. Participation was voluntary, and participants could refuse to continue at any time during the research period without consequences. Ethical approval was obtained from the Ethical Committee of the Affiliated Hospital of Jiangnan University (LS2021007). The study was conducted in accordance with the Declaration of Helsinki.

### 2.2. Measurement of Decision-Making Roles

The preferred and actual decision-making roles of ICU patients' family members were measured using the Simplified Chinese version of the Control Preference Scale (CPS) [16]. The test-retest reliability of the Simplified Chinese version was 0.82–0.87 [17]. The CPS consists of five options A–E, which are divided into two parts: preferred and actual. In this scale, A: I (prefer to) make the decision about which treatment I will receive; B: I (prefer to) make the final decision about my treatment after seriously considering my doctor's opinion; C: (I prefer that) my doctor and I share responsibility for deciding which treatment is best for me; D: (I prefer that) my doctor makes the final decision regarding which treatment will be used but seriously considers my opinion; E: I (prefer to) leave all decisions regarding treatment to my doctor. In both sections of the scale, options A and B are the active decision roles, option C is the cooperative decision role, and options D and E are the passive decision roles. The family members of ICU patients filled out both the preferred and actual role sections at the time of the survey.

### 2.3. Measurement of Anxiety and Depression

We used the Chinese version of Generalized Anxiety Disorder-7 (GAD-7) for anxiety screening and assessment of severity. The Chinese version of this scale includes seven questions, each scored from 0 to 3, where 0–4 points indicate no anxiety, 5–9 points indicate mild anxiety, 10–14 points indicate moderate anxiety, and 15–21 points indicate severe anxiety [18].

To measure symptoms of depression in family members, the Patient Health Questionnaire-9 (PHQ-9) was used for screening and severity assessment [19]. The scale consists of nine questions, each of which is scored from 0 to 3, where 0–4 points indicate no depression, 5–9 points indicate mild depression, 10–14 points indicate moderate depression, 15–19 points indicate moderate-to-severe depression, and 20–27 points indicate severe depression.

### 2.4. Data Collection

During the enrollment period, the ICU nursing staff initially identified eligible patients and approached their family members during their ICU visits to assess their willingness to meet with the investigator. The investigator explained the purpose and significance of this study to the families who agreed to participate. At 48–72 hours following ICU admission, family members' sociodemographic data were collected using a form, including gender, age, education, marital status, residence, medical bill payment method, monthly income, and relationship with the patient. The family members completed the surveys after signing the informed consent forms. Respondents were asked to fill out the questionnaires independently. Those who could not do this were assisted by the investigators, who read the survey to them item by item. The investigator then filled in responses on their behalf and completed each interview within 20–30 minutes. The investigators immediately returned the questionnaire to the respondents to verify that each question was answered.

### 2.5. Statistical Analysis

The data were statistically analyzed using the statistical software SPSS version 22.0 (IBM, Armonk, NY, USA). Descriptive statistics, such as frequencies, percentages, means, and standard deviations (SD), were used to describe participants' characteristics. The family members' responses to anxiety and depression were dichotomized into positive and negative screens for symptoms. Data were stratified according to the mismatch of family members' decision roles. Cross-tabular univariate analyses with chi-square or Fisher's exact tests were used to explore the relationship between the categorical variables and anxiety and depressive symptoms, and the kappa test was used to compare the consistency between the preferred and actual decision-making roles.

To explore factors associated with anxiety and depression symptom prevalence, binary logistic regression models were performed using anxiety and depression symptoms as dependent variables to adjust for potential confounders. Independent variables included those that were statistically significant in univariate analysis, the age of the family member, and the relationship with the patient. The age of the family member and the relationship with the patient were clinically recognized as factors affecting family members' anxiety and depression and were therefore included. In our analysis, gender was associated with depression symptoms, and mode of payment was associated with anxiety symptoms, including both variables. A *P* value of <0.05 was considered statistically significant.

## 3. Results

### 3.1. Characteristics of Family Members

In total, 223 family members completed the questionnaire and were enrolled in this study. The sociodemographic characteristics of the family members are shown in Table 1. The mean age of the family members was 50.92 ± 12.75 years. Family members over 50 years of age accounted for 48.0% of the participants, and female family members accounted for 65.5%. Family members with a junior high school education or less accounted for 48.4%, and the majority of family members lived in urban areas (80.7%). The payment method of medical bills was mainly urban basic medical care, including employee medical insurance (41.7%) and medical insurance for urban and rural residents (23.2%). Regarding income levels, 61.0% of families reported a monthly income >3000 China yuan (CNY). The relationships with the patient included spouse (31.4%), parent (3.1%), child (56.1%), sibling (2.2%), and other (7.2%).

### 3.2. Mismatch between the Preferred and Actual Decision-Making Roles of ICU Patients' Family Members

In the preferred and actual role groups of ICU patients' family members, the active role rates were 16.1% and 8.1%, the cooperative role rates were 49.3% and 31.4%, and the passive role rates were 34.5% and 60.5%, respectively (Table 2). The incidence of mismatch between preferred and actual roles was 43.0% in medical decision-making for ICU patients' families, and the consistency between their preferred and actual decision-making roles was poor (kappa = 0.309, *P* < 0.001). The results showed that the degree of preferred decision-making participation was higher than the actual participation level.

### 3.3. Symptoms of Anxiety and Depression

The incidence of anxiety was 71.7% among family members of ICU patients, and the rates of mild anxiety, moderate anxiety, and severe anxiety were 40.8%, 22.4%, and 8.5%, respectively. The incidence of depression was 73.1% among family members of ICU patients, and the rates of mild depression, moderate depression, and moderate to severe depression were 37.7%, 31.4%, and 4.0%, respectively. Univariate analysis showed that education level, residence, and monthly income were associated with the incidence of anxiety and depression in ICU family members (*P* < 0.05). Female family members had higher depression rates than males (77.4% vs. 64.9%; *P* = 0.046). There was no significant correlation between incidence of anxiety and gender (*P* > 0.05), but the incidence of anxiety was associated with mode of payment (*P* = 0.003). There were no associations of either anxiety or depression rates with family member's age, marital status, or the relationship with patients (*P* > 0.05) (Table 3).

### 3.4. The Associations of Role Mismatch in Decision-Making with Anxiety and Depression

**Figure 1:**
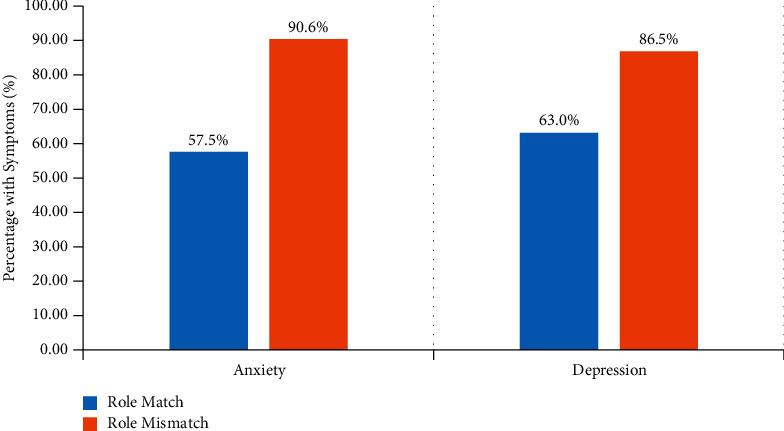
Psychological symptoms of family members were stratified by whether the decision role was matched. Comparing the percentage of role-matched and role-unmatched family members experiencing depression or anxiety symptoms; all *P* values < 0.001.

Family members with mismatched decision-making roles had significantly higher rates of symptoms of anxiety (90.6% vs. 57.5%; *P* < 0.001) and depression (86.5% vs. 63.0%; *P* < 0.001) (Figure 1). In a binary logistic regression model adjusting for family members' gender, age, education, residence, payment method, monthly income, and relationship with the patient, role mismatch in decision-making remained independently associated with more symptoms of anxiety (odds ratio (OR) = 12.821, 95% confidence interval (CI): 5.004–32.845, *P* < 0.001) and depression (OR = 5.224, 95% CI: 2.343–11.648, *P* < 0.001) (Table 4).

## 4. Discussion

Nearly half of the ICU patients' family members (43%) experienced a mismatch between the preferred and actual roles in decision-making. The results of a study in the United States showed that 13.7% of the family members of ICU patients experienced a mismatch between their preferred and actual roles [13]. Our findings showed a significantly higher mismatch rate than that in the United States, indicating that the current mismatch in the decision-making role of ICU patients' family members is more serious in China and needs to be considered by medical staff. In China, the large population number, shortage of medical resources, and busy treatment activities have led to insufficient communications between medical staff and their families [8]. Medical staff do not fully understand the feelings of family members about their role in decision-making [20], which affects family members' experiences of mismatch in decision-making.

We examined the relationship between decision-making role mismatch and anxiety and depression in ICU family members. The anxiety and depression symptoms of family members who experienced decision role mismatch were significantly higher than those of family members who matched their decision role. Importantly, decision role mismatch remained independently associated with more anxiety and depressive symptoms after adjustment for various important factors, which was similar to a previous report [13]. Improved satisfaction was reported among family members of ICU patients if the preferred and actual decision-making roles matched [21]. These results show that by providing actual roles that are consistent with the preferred roles of the family members, the development of their psychological symptoms can be affected, and family satisfaction can be improved.

The China's Fourth National Health Service Survey showed that patients and their families have increased demand for participation in decision-making [22]. Our data showed that the preferred decision-making roles of ICU patients' family members were mainly cooperative (49%), followed by passive (35%) and active (16%). This is consistent with previous findings indicating that ICU patients' family members have good awareness of decision-making participation and are willing to be involved [23, 24]. In this study, the ICU patients' family members preferred the cooperative decision-making role, which was adapted to the shared decision-making currently recommended as an ICU medical decision-making model [25]. This shows that ICU patients' family members are willing to share decision-making responsibilities with medical staff.

Our study showed that ICU patients' family members preferred to play a cooperative role in the decision-making process (49%), while the actual decision-making process was mainly passive (61%). This indicated that ICU patients' family members had strong willingness to participate in patients' medical decisions [26], but there was a large difference in the actual decision-making role experience. Previous studies have shown that ICU patients' family members face significant challenges in the decision-making process [27, 28], which may affect the actual decision-making role. Being forced to make decisions in a short period of time can be stressful for families who are unsure as to whether they will make the best decision [27]. Currently, the effect of doctor–patient communication is poor in the ICU [29], resulting in insufficient understanding of the patient's condition and treatment plan by family members, which has a negative impact on the confidence of family members participating in decision-making [28]. The opinions of other family members increase the burden and complexity of the primary decision-maker role, influencing the selection of actual decision role [27].

Our findings further elucidate the current status and impact of family member involvement in medical decision-making for ICU patients. Based on the results, we recommend that ICU medical staff adopt a patient and family-centered communication model in medical decision-making, invite family members to participate in daily ward rounds, and discuss the patient's condition and care plan together. This could meet the information needs of family members and provide family members with the opportunity to participate in medical decision-making and further understand the personal characteristics, decision-making needs, preferences, and values of family members, thus directly affecting the outcome and satisfaction of medical decision-making [30–32]. Nurses can also play an active role in monitoring and improving the decision-making process of family members, understanding their needs and providing support [33]. By arranging designated nursing staff to communicate with family members continuously, nurses can have a more comprehensive understanding of their patients' families.

Our study had several limitations. First, this was a single-center study with the convenient sampling method and a relative small sample size. Future large-scale multicenter studies are needed to validate the results from this study. Second, the psychological questionnaire we utilized can only measure the symptoms of anxiety and depression and cannot be used for disease diagnosis. Finally, this study used a scale to evaluate the decision-making participation of ICU family members, which makes it possible to ignore the specific decision-making participation experience of family members. Thus, it is necessary to specifically explore the decision-making participation process of ICU families in the future.

## 5. Conclusions

We demonstrated that decision-making role mismatch is independently associated with more symptoms of anxiety and depression in family members of ICU patients. Medical staff should pay more attention to the decision-making preferences of patients' family members to provide personalized decision-making participation for family members of ICU patients. Development of an optimized system for medical decision-making participation for ICU patients' families is needed to meet the participatory needs of patients' families.

## Figures and Tables

**Table 1:** Sociodemographic characteristics of family members.

Characteristics	*n* (%)
Mean age (years)	50.92 ± 12.75
20–30 years	13 (5.8)
31–40 years	35 (15.7)
41–50 years	68 (30.5)
51–60 years	61 (27.4)
>60 years	46 (20.6)
Gender	
Male	77 (34.5)
Female	146 (65.5)
Education	
Primary school or less	29 (13.0)
Junior high school	79 (35.4)
Senior high school	52 (23.3)
Junior college	38 (17.0)
University or above	25 (11.2)
Marital status	
No	6 (2.7)
Yes	217 (97.3)
Residence	
City	180 (80.7)
Town	6 (2.7)
Rural area	37 (16.6)
Payment method	
Own expense	14 (6.3)
Public expense	4 (1.8)
Employee medical insurance	145 (65)
Medical insurance for urban and rural residents	58 (26)
Commercial medical insurance	2 (0.9)
Monthly income	
<1000 CNY	21 (9.4)
1000–3000 CNY	66 (29.6)
>3000 CNY	136 (61.0)
Relationship	
Spouse	70 (31.4)
Parent	7 (3.1)
Child	125 (56.1)
Sibling	5 (2.2)
Others	16 (7.2)

SD: standard deviation; CNY: China yuan, Chinese currency.

**Table 2:** Preferred and actual roles of ICU patients' families in decision participation.

Item	Actual decision role, *n* (%)			Total, *n* (%)
	Active	Cooperative	Passive	
Preferred decision role				
Active	16 (7.2)	14 (6.3)	6 (2.7)	36 (16.1)
Cooperative	2 (0.9)	45 (20.2)	63 (28.3)	110 (49.3)
Passive	0 (0)	11 (4.9)	66 (29.6)	77 (34.5)
Total	18 (8.1)	70 (31.1)	135 (60.5)	223 (100)

**Table 3:** Univariate analysis of ICU patients' family members' anxiety and depression.

Characteristics	Anxiety			Depression		
	Yes, *n* (%)	No, *n* (%)	*P* Value	Yes, *n* (%)	No, *n* (%)	*P* Value
Age			0.143^*∗*^			0.194^*∗*^
20–30 years	11 (84.6%)	2 (15.4%)		11 (84.6%)	2 (15.4%)	
31–40 years	27 (77.1%)	8 (22.9%)		26 (74.3%)	9 (25.7%)	
41–50 years	44 (64.7%)	24 (35.3%)		43 (63.2%)	25 (36.8%)	
51–60 years	40 (65.6%)	21 (34.4%)		45 (73.8%)	16 (26.2%)	
>60 years	38 (82.6%)	8 (17.4%)		38 (82.6%)	8 (17.4%)	
Gender			0.310			0.046
Male	52 (67.5%)	25 (32.5%)		50 (64.9%)	27 (35.1%)	
Female	108 (74.0%)	38 (26.0%)		113 (77.4%)	33 (22.6%)	
Education			0.039			0.040
Primary school or less	26 (89.7%)	3 (10.3%)		27 (93.1%)	2 (6.9%)	
Junior high school	59 (74.7%)	20 (25.3%)		58 (73.4%)	21 (26.6%)	
Senior high school	36 (69.2%)	16 (30.8%)		38 (73.1%)	14 (26.9%)	
Junior college	26 (68.4%)	12 (31.6%)		26 (68.4%)	12 (31.6%)	
University or above	13 (52.0%)	12 (48.0%)		14 (56.0%)	11 (44.0%)	
Marital status			1.000^*∗*^			1.000^*∗*^
No	5 (83.3%)	1 (16.7%)		5 (83.3%)	1 (16.7%)	
Yes	155 (71.4%)	62 (83.3%)		158 (72.8%)	59 (27.2%)	
Residence			0.001^*∗*^			0.031^*∗*^
City	120 (66.7%)	60 (33.3%)		126 (70%)	54 (30%)	
Town	5 (83.3%)	1 (16.7%)		4 (66.7%)	2 (33.3%)	
Rural area	35 (94.6%)	2 (5.4%)		33 (89.2%)	4 (10.8%)	
Payment method			0.003^*∗*^			0.093^*∗*^
Own expense	13 (92.9%)	1 (7.1%)		12 (85.7%)	2 (14.3%)	
Public expense	3 (75.0%)	1 (25.0%)		2 (50.0%)	2 (50.0%)	
Employee medical insurance	93 (64.1%)	52 (35.9%)		100 (69.0%)	45 (31.0%)	
Medical insurance for urban and rural residents	50 (86.2%)	8 (13.8%)		48 (82.8%)	10 (17.2%)	
Commercial medical insurance	1 (50.0%)	1 (50.0%)		1 (50.0%)	1 (50.0%)	
Monthly income			0.009			0.012
<1000 CNY	20 (95.2%)	1 (4.8%)		21 (100.0%)	0 (0.0%)	
1000–3000 CNY	51 (77.3%)	15 (22.7%)		48 (72.7%)	18 (27.3%)	
>3000 CNY	89 (65.4%)	47 (34.6%)		94 (69.1%)	42 (30.9%)	
Relationship			0.261^*∗*^			0.145^*∗*^
Spouse	54 (77.1%)	16 (22.9%)		56 (80.0%)	14 (20.0%)	
Parent	7 (100.0%)	0 (0.0%)		7 (100.0%)	0 (0.0%)	
Child	85 (68.0%)	40 (32.0%)		87 (69.6%)	38 (30.4%)	
Sibling	3 (60.0%)	2 (40%)		3 (60.0%)	2 (40.0%)	
Others	11 (68.8%)	5 (31.3%)		10 (62.5%)	6 (37.5%)	

CNY: China yuan, Chinese currency. ^*∗*^Fisher's exact test.

**Table 4:** Binary logistic regression analysis of factors affecting anxiety and depression in ICU patient family members.

Item	Anxiety			Depression		
	OR	95% CI	*P* Value	OR	95% CI	*P* Value
Decision-making role mismatch (no as reference)						
Yes	12.821	5.004–32.845	<0.001	5.224	2.343–11.648	<0.001
Age (20–30 years as reference)						
30–40 years	1.209	0.136–10.744	0.865	0.991	0.136–7.244	0.993
40–50 years	0.342	0.044–2.634	0.303	0.335	0.052–2.142	0.248
50–60 years	0.219	0.028–1.738	0.151	0.407	0.061–2.733	0.355
>60 years	0.364	0.034–3.867	0.402	0.405	0.046–3.601	0.418
Gender (male as reference)						
Female	2.155	0.923–5.031	0.076	2.884	1.303–6.383	0.009
Education (primary school or less as reference)						
Junior high school	1.014	0.196–5.254	0.987	0.451	0.080–2.534	0.366
Senior high school	1.046	0.185–5.916	0.959	0.617	0.103–3.708	0.598
Junior college	0.531	0.084–3.345	0.500	0.338	0.051–2.219	0.258
University or above	0.234	0.030–1.842	0.168	0.178	0.023–1.385	0.099
Residence (city as reference)						
Town	3.475	0.271–44.606	0.339	0.909	0.103–8.046	0.932
Rural area	5.434	0.815–36.248	0.080	2.672	0.533–13.402	0.232
Payment method (own expense as reference)						
Public expense	0.155	0.005–4.976	0.292	0.129	0.008–2.040	0.146
Employee medical insurance	0.244	0.026–2.317	0.219	0.673	0.116–3.891	0.658
Medical insurance for urban and rural residents	0.416	0.039–4.441	0.468	0.767	0.126–4.674	0.774
Commercial medical insurance	0.038	0.000–3.463	0.156	0.105	0.002–6.573	0.286
Monthly income (<1000 CNY as reference)						
1000–3000 CNY	0.353	0.026–4.751	0.433	0.000	0.000–0.000	0.998
>3000 CNY	0.275	0.018–4.275	0.357	0.000	0.000–0.000	0.998
Relationship (spouse as reference)						
Parent	488741207.900	0.000–0.000	0.999	471881417.100	0.000–0.000	0.999
Child	0.528	0.197–1.416	0.204	0.690	0.263–1.810	0.451
Sibling	0.396	0.042–3.751	0.419	0.198	0.018–2.180	0.186
Others	1.039	0.196–5.509	0.965	0.644	0.138–0.644	0.577

OR: odds ratio; CI: confidence interval; CNY: China yuan, Chinese currency.

## Data Availability

The data used to support the findings of this study are available from the corresponding author upon request.
